# Family Thriving During COVID-19 and the Benefits for Children’s Well-Being

**DOI:** 10.3389/fpsyg.2022.879195

**Published:** 2022-05-12

**Authors:** Lindsey C. Partington, Meital Mashash, Paul D. Hastings

**Affiliations:** ^1^Department of Human Ecology, College of Agricultural and Environmental Sciences, University of California, Davis, Davis, CA, United States; ^2^Center for Mind and Brain, College of Letters and Science, University of California, Davis, Davis, CA, United States; ^3^Osher Center for Integrative Health, University of California, San Francisco, San Francisco, CA, United States; ^4^Department of Psychology, University of California, Davis, Davis, CA, United States

**Keywords:** well-being, COVID-19, resilience, thriving, family functioning

## Abstract

Although the COVID-19 pandemic has raised deserved concern regarding adverse impacts on parents’ and children’s mental health, regulations like “sheltering-in-place” may have afforded parents novel opportunities to foster positive family connections, thereby bolstering well-being. Using latent profile analysis (LPA), we (a) distinguished family thriving during shelter-in-place (May-June 2020) from other patterns of family functioning, (b) tested potential predictors of family functioning profiles, and (c) examined if family thriving predicted subsequent child adjustment (September–October 2020). 449 parents in two-parent U.S. families with children aged 2–18 years completed online surveys assessing (a) parent–child relationship quality, parents’ positive psychological adjustment, children’s emotional well-being, and parenting efficacy and satisfaction as family functioning indicators, (b) financial, marital, parental psychosocial assets, and child (age, gender, and temperament) predictors of family functioning, and (c) child adjustment. LPA identified four family functioning profiles: Thriving, Managing, Struggling, and Distressed. Thriving families evinced higher scores on all functioning indicators. Logistic regressions revealed that parents in Thriving families reported significantly lower financial anxiety, less dissatisfaction with partner’s help, less child emotionality, and greater use of cognitive reappraisal, as well as more positive child adjustment in Fall 2020. These findings underscore the multidimensional nature of coping and well-being during COVID-19. Utilizing these levers to promote mental health in families languishing during comparable future crises could promote resilience, thereby protecting children’s well-being.

## Introduction

The COVID-19 pandemic produced myriad changes within family systems, with shelter-in-place, remote-schooling and remote-work conditions introducing a period of heightened risk for parenting stress and parent-child conflict (Brown et al., 2020; Cluver et al., 2020; Fong and Iarocci, 2020; Russell et al., 2020). These multiple, unexpected challenges may have eroded parents’ emotional well-being and mental health, compromising their childrearing skills (Brown et al., 2020; Russell et al., 2020). Yet, these same conditions also may have afforded parents novel opportunities to foster positive family connections that could bolster their own and their children’s well-being (Chu et al., 2021; McArthur et al., 2021). Families evincing good parent and child mental health, positive parent-child relationship quality, and parent efficacy in the context of the pandemic could be seen as thriving despite the challenges. Yet, most research has been focused on the family stresses and challenges incurred by the pandemic, including more harsh parenting, worse mental health and more psychological distress (Park et al., 2020; Patrick et al., 2020; Pierce et al., 2020; Russell et al., 2020; Feinberg et al., 2021). Such studies do not provide insight into which families adjusted well despite the challenges of COVID-19, what factors predicted this family thriving, and whether family thriving conferred lasting benefits for children. Examining these gaps could elucidate ways to support families during continued pandemic conditions and future global health crises (Masten and Motti-Stefanidi, 2020).

**FIGURE 1:**
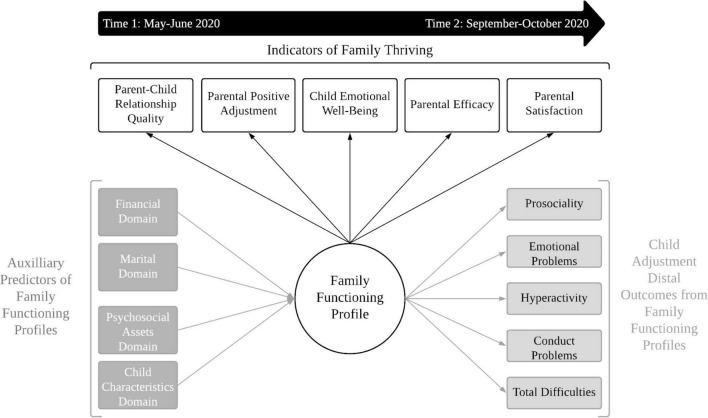
Conceptual figure of study aims. Parent–child relationship quality, parental positive adjustment, child emotional well-being, parental efficacy, and parental satisfaction are expected to underlie multiple patterns of family functioning during the early stages of the pandemic (May–June 2020), with one pattern distinguishing a family thriving profile. Multiple factors within financial, marital, psychosocial assets, and child characteristic domains are expected to distinguish family thriving from other family functioning profiles. Finally, family functioning profiles early in the pandemic are expected to influence children’s subsequent adjustment as the pandemic continued (September–October 2020).

The present study explored patterns of family functioning in the months shortly after shelter-in-place was instated across most of the United States (May–June 2020). Parents’ adjustment likely serves as a conduit by which pandemic disruptions infiltrate the family system via family interactions, with parent’s positive adjustment buffering their children against pandemic stressors and promoting children’s mental and emotional health during shelter-in-place (Prime et al., 2020). Person-centered approaches were used to model the heterogeneity of *individual families*, identifying subgroups of families that could be distinguished on the basis of similarity of behavioral patterns or characteristics during the pandemic (Lanza and Cooper, 2016). Additionally, we aimed to identify key financial, marital, and psychosocial assets, and child characteristics, that distinguished between family functioning profiles, and to assess the extent to which family functioning profiles prospectively predicted child adjustment. A conceptual model of the study goals is presented in Figure 1.

### Dynamics of Family Functioning During COVID-19

Beginning in March 2020, the majority of the U.S instituted “shelter-in-place” measures resulting in massive closures of schools, childcare centers, and non-essential businesses coupled with a transition to remote work and education (CDC, 2022). Although initially expected to last a few weeks, these pandemic conditions continued for months. Families’ daily lives and routines were unequivocally disrupted within a context of pervasive global stress, fear, and anxiety surrounding COVID-19 (Feinberg et al., 2021; McCoy et al., 2021), eliciting extensive concerns early in the pandemic regarding the impacts of these unusual conditions on family welfare and children’s mental health (Cluver et al., 2020; Lee, 2020; Prime et al., 2020). Globally, psychological distress increased, and this distress persisted, with meta-analytic evidence suggesting that, for children and adolescents, prevalence of depression doubled in the wake of COVID-19 and remained high as the pandemic continued (Racine et al., 2021). Further, the pandemic did not affect families equally; families that were marginalized and economically disadvantaged before the pandemic experienced disproportionate hardship and were more severely impacted (Chen et al., 2021).

Acknowledging the profound difficulties that many families have experienced, the pandemic may have also presented novel opportunities for family connection. Both public (Wilson, 2020) and empirical works (Günther-Bel et al., 2020) suggested that not all families were detrimentally impacted by the pandemic. For some, family bonds appeared to have been bolstered due to increased time spent together during shelter-in-place. Across multiple countries, a minority (10–29%) of parents and children (14%) reported feeling that their families had increased closeness and cohesion and decreased conflict in the first few months of the pandemic (Brown et al., 2020; Chavez et al., 2021; Chu et al., 2021). Notably, Chavez et al. (2021) reported that multi-person households with children evinced greater cohesion despite also evincing greater social vulnerability (e.g., unemployment, overcrowding, low educational attainment, etc.), compared to multi-person households without children. This may suggest that families with children possess unique sources of strength and resilience (Turliuc et al., 2013). Moreover, emerging evidence suggests that close family relationships buffered against pandemic distress, both in children (McArthur et al., 2021) and parents (Neubauer et al., 2021).

Informed by family resilience perspectives (Masten, 2015; Walsh, 2015), family systems theory (Cox and Paley, 1997) and other parenting models (Bugental and Johnston, 2000; Bornstein et al., 2018), as applied to the context of pandemic conditions (Masten and Motti-Stefanidi, 2020; Prime et al., 2020), we examined five indicators of family thriving. Specifically, we measured parent-child relationship quality, parents’ positive psychological adjustment, children’s emotional well-being, parental satisfaction, and parental efficacy, during the third month of shelter-in-place. Family-level promotive factors like close and cohesive relationships (Masten and Palmer, 2019) contribute to better-than-expected family adjustment during and after largescale disasters (Masten and Motti-Stefanidi, 2020; Prime et al., 2020). Parental satisfaction and efficacy during the shelter-in-place period reflect the parents’ sense that they are effectively managing family needs (Jones and Prinz, 2005), which could promote supportive parenting (Bornstein et al., 2018). Close relationships, parental satisfaction and parental efficacy likely are influenced by, and contribute to, parents’ and children’s psychological well-being (Prime et al., 2020). Hence, it is plausible that, collectively, these five indicators may serve to demarcate those families who thrived within the challenging and unusual context of the early months of the pandemic.

Using latent profile analysis (LPA) we can model how these family functioning indicators co-occur within a family system to create distinct family functioning profiles, by which the family becomes the level of analysis (Bergman and Trost, 2006). As a person-centered approach, LPA can provide an integrative perspective on the global family environment during the early stages of the pandemic. By modeling the heterogeneity of families as opposed to variability within a construct or variable, person-centered analyses enable us to characterize families more holistically in terms of their specific response patterns to the pandemic, identifying subgroups of families with similar behavioral patterns or characteristics that are also distinct from other subgroups (Lanza and Cooper, 2016). That is, the family becomes the level of analysis encompassing a system of patterned, coinciding responses and processes (Bergman and Trost, 2006). Identifying thriving and other subgroups of families provides a multidimensional view of family functioning and coping during the pandemic, which may offer novel insights for targeted points of family intervention during a global health crisis with consideration for multifactorial rather than one-dimensional aspects of family functioning.

Just as person-centered methodologies have been effective for furthering our understanding of how specific combinations of contextual risk relate to children’s behavioral development beyond cumulative risk exposure (Lanza and Cooper, 2016), these same approaches can potentially be applied to identifying profiles of thriving. While LPA has frequently and effectively been used in studying patterns of risk and adversity, it has been less often applied to the study of more general family function. Previous research utilizing person-centered methodologies in family well-being have consistently found a latent subgroup of individuals who are doing well despite stress exposure, including maternal well-being prior to COVID-19 (Recksiedler et al., 2021), Chinese adolescents with largely improving family and social relationship during COVID-19 (Shen et al., 2021), and international families (with and without children) brought together despite COVID-19 social distancing regulations (Chavez et al., 2021). While these studies have found a range of functioning profiles across myriad indicators, their findings suggest that thriving is consistently evident in the wake of adversity. The current investigation expands upon previous research by focusing on U.S. families with children living at home, examining what factors predict U.S. family functioning profiles, and how these patterns of family functioning early in the pandemic relate to children’s later adjustment.

### Antecedents of Family Functioning

It is also important to consider which factors predict thriving versus less positive family adjustment. The capacity for the family system to effectively respond to pandemic conditions may be impacted by both pandemic-specific influences (e.g., job loss, risk of infection) and pre-existing characteristics (e.g., coping skills; Prime et al., 2020). The present study focused on the domains of financial impact, marital dynamics, coping skills, and child characteristics. These domains encompass pandemic-induced disruptions to the family system (e.g., financial difficulties) as well as pre-existing characteristics (e.g., child temperament) that may have shaped the cohesion and functioning of families during the stressful context of the pandemic.

#### Financial Domain

Three factors were examined within the financial domain: Pre-pandemic income (2019 income per capita), financial difficulty (difficulty paying bills or covering essential expenses), and financial anxiety (worry about potentially not having sufficient financial resources). Pre-existing vulnerabilities, such as pre-pandemic economic hardship, may impede a family’s ability to cope with pandemic conditions (Prime et al., 2020). Less financially well-off families being more adversely affected by the pandemic and associated public health regulations accompanied with heightened parenting concerns (Chen et al., 2021; Feinberg et al., 2021; Schmeer et al., 2021).

Shelter-in-place and other public health efforts introduced a national economic threat (Bartik et al., 2020) that contributed to heightened financial stress (Mann et al., 2020) and decreased ability to afford basic needs (Roll and Despard, 2020). Early studies of pandemic effects showed that overall financial stress compromised family well-being during shelter-in-place (Gassman-Pines et al., 2020). From the perspective of the family stress model (Conger and Elder, 1994; Conger and Conger, 2002), both pandemic-induced financial difficulty in affording basic needs and financial anxiety about future expenses would have been likely to result in heightened marital discord and harsh parenting, eroding family well-being.

#### Marital Domain

Two factors were explored within the marital domain: general marital quality (not specific to the pandemic) and satisfaction with partner’s help in the household. Largescale disasters may force couples to depend on each other more heavily for support as other social partners (e.g., friends, extended family) are less available (Cohan, 2010), and during the pandemic, inaccessible. During shelter-in-place, marital tension may have been exacerbated and marital quality degraded for some couples (Karney et al., 2005) but for others, high quality marital relationships may have been maintained, further protecting the family system against the effects of external risks (Merz et al., 2014; Walsh, 2015). Marital difficulties prior to major life events may contribute to heightened stress and new difficulties within the family system, as evident from natural disaster research (Cohan, 2010). Consequently, while preliminary evidence suggests that marital quality may change as a function of pandemic conditions (Chu et al., 2021), there is robust literature suggesting that marital quality prior to significant life events may buffer the family against threats to well-being as spouses in higher quality relationships act as a united front whereas lower quality relationships may exacerbate the effects of newly induced pandemic stress as spouses cannot depend on each for support.

The transition to work-from-home coupled with remote-schooling and childcare closures disproportionately impacted working women (Rivera et al., 2020; Yavorsky et al., 2021). Being required to do disproportionately more domestic labor than one’s partner was associated with women – but not men – reporting worse relationship quality relative to pre-pandemic levels (Waddell et al., 2021). In dual-earner U.S. families, a “wife does it all” approach to managing household labor was associated with decreased family well-being and wives’ increased psychological distress, whereas an egalitarian division of labor was associated with more optimal family functioning (Shockley et al., 2020). Hence, a parent’s satisfaction with a partner’s contribution to domestic duties likely impacts family well-being.

#### Psychosocial Assets Domain

Two factors were examined within the psychosocial assets domain: parent’s active coping skills and use of cognitive reappraisal. Active coping encompasses the use of psychosocial resources to address problems (Carroll, 2013), and is a robust predictor of thriving at the individual and family levels (Walsh, 2015; Masten and Motti-Stefanidi, 2020). Parents’ coping abilities facilitate children’s understanding and sense of coherence during disasters (Eriksson and Lindstrom, 2005), foster a positive outlook within the family system (Walsh, 2015), and may socialize effective coping in children (Gottman et al., 1996; Eisenberg et al., 1998). Effective emotion regulation strategies likely also contribute to parents’ abilities to cope with pandemic conditions (Prime et al., 2020). Cognitive reappraisal is a regulatory strategy in which the individual re-conceptualizes an evocative situation to change its emotional meaning (Gross, 2002). It may enable parents to reconceptualize pandemic stressors as opportunities for growth, thereby facilitating family connection and positive interactions. An intervention study with German families found that cognitive reappraisal training decreased parenting stress during COVID-19 (Preuss et al., 2021). Thus, parents’ active coping skills and their use of cognitive reappraisal may each promote optimal family functioning during COVID-19.

#### Child Characteristics Domain

For child characteristics, we examined the effects of child age, child gender, and child temperament in distinguishing family functioning profiles. During shelter-in-place, younger children are more dependent on parents (Feinberg et al., 2021), contributing to increased parenting stress (Giannotti et al., 2021), and more work absences due to childcare obligations (Fong and Iarocci, 2020). With regard to gender, a meta-analysis showed that girls experienced more severe depression and anxiety than boys during the early stages of the pandemic (Racine et al., 2021), which could further challenge parents. Children with highly emotional temperaments exhibit more negative affect like anger, fear, and sadness (Rothbart et al., 1994), and have more stressed parents (Östberg and Hagekull, 2000). Potentially, temperamentally emotional children may have had greater difficulty adjusting to the pandemic conditions, contributing to an emotionally charged and frustrating family climate. During COVID-19, children’s negative emotionality was found to be associated with both parent and child anxiety and distress during the first 5 months of the pandemic (Zhou et al., 2021). Thus, we examined child age, gender and emotional temperament as predictors of patterns of family functioning in the wake of shelter-in-place.

### Prospective Impact of Acute Pandemic Response

As COVID-19 continues and new variants arise, and evidence mounts for the adverse effects of pandemic living conditions on children’s mental health (Bignardi et al., 2021; Creswell et al., 2021), there is growing need to examine the factors that may buttress children’s well-being and healthy adjustment (Wade et al., 2020). Effective family functioning during the first few weeks of shelter-in-place has been associated with children’s concurrent positive adjustment (Chu et al., 2021; McArthur et al., 2021), with benefits for children’s adjustment potentially persisting for several months (Hastings et al., 2021). How families initially responded to and coped with COVID-19 may have had persisting influences on children’s emotional and behavioral trajectories as the pandemic continued. Family thriving could have better equipped children to cope effectively and maintain their well-being.

### The Current Investigation

The purpose of the current investigation was to identify families that were thriving in the first months of the pandemic, examine what distinguished them from other families, and test whether family thriving conveyed lasting benefits for children’s mental health. (H1) We hypothesized a profile of family thriving, characterized by high-quality parent-child relationships, high levels of parental satisfaction and feelings of efficacy, and positive psychological adjustment from both parent and child, would be distinguishable from other profiles of family functioning. (H2) We expected that the thriving family profile would be predicted by fewer financial impacts, greater coping and cognitive reappraisal skills, greater marital support, and less child temperamental emotionality. (H3) We predicted that, compared to families with other patterns of functioning, thriving families in Spring 2020 would have children who evinced better adjustment and well-being in Fall 2020, as the pandemic continued.

## Materials and Methods

### Participants

From May 26 through June 18, 2020, advertisements for an online study to better understand parenting and family functioning during the pandemic were placed on social media (e.g., Facebook parenting groups) and disseminated through university newsletters and participant recruitment webpage venues in the United States. Additional recruitment advertisements were targeted to communities with highly diverse populations in an attempt to increase sample diversity, but with very limited effectiveness. Recruitment criteria were being a parent aged 18 years or older, having at least one child between the ages of 2–18 years who was living at home, ability to read and write in English, and access to an electronic device with internet capabilities. At Time 1, 492 parents participated. At Time 2, from September 3 to October 3, 2020, families who had consented to being contacted for future research (*N* = 232) were asked to complete a short follow-up survey.

Because of the planned examination of the Marital Domain in relation to profiles of family functioning, the current analysis was restricted to two-parent families (*n* = 454). Five families did not complete any measures for the family functioning indicators and were not used for the LPA, leaving a final sample size of 449 families. The sample was predominantly educated (49.400% post-graduate degree), White mothers (89% female, 70% White) in their late 30s (*M*_*Parent Age*_ = 38.040, *SD*_*Parent Age*_ = 6.327) living in two-child households (*M*_*Number of Children*_ = 2.000, *SD*_*Number of Children*_ = 0.921). For several questions in the survey, parents with multiple children were instructed to focus on one child and to answer the questions in relation to that specific child (*M*_*Child Age*_ = 7.019, *SD*_*Child Age*_ = 4.067, 46% female). Families had a median household income of $100,000–$149,999 for 2019 (*Range:* less than $10,000 to greater than $150,000; 33% with income greater than $150,000) and a median income per capita of $31,250 (*Range:* $833–$87,500). 41% of families reported an essential worker living in the house, of which 12% were healthcare workers. Time 2 data were collected from 219 (48%) of these parents; again, most respondents were educated (51.60% post-graduate degree), White mothers (92% female, 71% White) in their late 30s (*M*_*Parent Age*_ = 38.051, *SD*_*Parent Age*_ = 5.644). Our study sample had a higher proportion of adults with a post-graduate degree and higher household income in comparison to national demographics in 2019. In the general U.S. population, 32% of U.S. adults (25 years or older) had a post-graduate degree (McElrath and Martin, 2021) and median household income for a 2-parent family was $101,417 (Shrider et al., 2021).

### Procedure

The UC Davis IRB Administration approved the project under IRB ID 1604542 and determined the project as Exempt 2 status. Participants completed the study using Qualtrics, an online data collection tool (Qualtrics, 2020). Prior to completing the survey, participants viewed an online informed consent page describing the purpose of the survey and the approximate duration of the survey. Additionally, the informed consent page stated that participation was voluntary, that participants could withdraw from the study or decline to answer questions without penalty or loss of compensation, and that they could opt-in to being contacted for future research opportunities. Participants who consented to participation were then directed to the online survey. Participants who declined participation were directed to a page thanking them for their time and interest in research. Identifying information (e.g., email addresses for electronic compensation) was removed from the main dataset and stored in a separate dataset to allow for electronic compensation for study participation and continued contact with participants who consented to being contacted for future research.

At Time 1 (T1), participants provided consent, and reported on their demographics, pandemic-specific economic hardship, marital quality, social support, family functioning, parenting behavior, and parent’s and children’s mental health and adjustment during the pandemic. Parents received a $15 Amazon gift card for their participation. The survey took participants approximately 45 min to complete. At Time 2 (T2), parents completed an additional consent form and then completed questionnaires regarding pandemic-specific economic hardship, marital quality, family functioning, and parent and child adjustment. The T2 survey took participants approximately 10–15 min to complete. Parents were entered into a raffle to receive one of four $25 Amazon gift cards as compensation for their participation.

### Measures

To minimize participant burden while capturing a wide breadth of variables, some measures were reduced to only contain key items with high face validity. This decision impacted the following variables: parenting efficacy, parenting satisfaction, financial anxiety, marital quality, and child temperament.

#### Family Functioning Indicators

##### Parent–Child Relationship Quality

Relationship quality was assessed with 1 item from the Coronavirus Health and Impact Survey (CRISIS, Nikolaidis et al., 2021). Using a 5-point, Likert-type scale, parents reported if the quality of their relationship with their child had changed compared to the pre-pandemic period (*“Since the pandemic began, has the quality of your relationship between you and ‘focal child’ (the child you identified at the start of the survey) changed?”, 1 = Yes, it’s a lot worse, 5 = Yes, it’s a lot better*).

##### Parent Positive Adjustment

Parent’s positive adjustment to the pandemic was measured with the 5-item Revised Mental Health Inventory-5 (MHI-5, Berwick et al., 1991). The measures include two items assessing positive adjustment (*e.g., “Since the pandemic began, how much of the time have you been a happy person?”)* and three items assessing negative adjustment (e.g., “… *a very nervous person*?”). Parents were asked to rate the frequency of which they had experienced each statement using a 6-point, Likert-type scale (*1 = none of the time, 6 = all the time*). Responses to negative adjustment items were reversed scored and a mean score with all five items was calculated with higher scores indicating better adjustment to the pandemic (α = 0.791).

##### Parenting Efficacy

We selected and adapted three items from the Parenting Sense of Competence scale (Johnston and Mash, 1989) to measure parental efficacy during the pandemic. Parents rated the extent to which they agreed with each statement [e.g., *“Being a parent during the pandemic is manageable, and any problems are easily solved,” “I honestly believe I have all the skills necessary to be a good parent to my child(ren) during the pandemic”]* using a 6-point, Likert-type scale (*1 = strongly disagree, 6 = strongly agree*). Responses to one negatively worded item (*“Being a parent during the pandemic makes me tense, anxious, and frustrated”*) were reverse scored, and a mean score with all three items was calculated with higher scores indicating greater feelings of efficacy (α = 0.722).

##### Parenting Satisfaction

We adapted one item from the Kansas Parental Satisfaction Scale (James et al., 1985) to measure parenting satisfaction during the pandemic. Parents were asked to rate the extent to which they were satisfied with themselves as parents during the pandemic using a 7-point, Likert-type scale (*“How satisfied are you with yourself as a parent during the pandemic?”, 1 = Very Dissatisfied, 7 = Very Satisfied*).

##### Child Emotional Well-Being

Parents rated children’s emotional well-being during the pandemic using one-item from the CRISIS (”How would you rate your child’s overall mental and emotional health now?”; Nikolaidis et al., 2021) using a 5-point scale (*1 = Poor, 5 = Excellent*).

#### Predictors of Family Functioning Profiles

##### Financial Domain

###### Income per Capita

Income per capita was calculated by dividing self-reported annual household income from 2019 by the reported number of people living in the household. Next, we divided the resulting number by 10,000 to create values on a comparable scale to other measures. Higher scores reflect higher income per capita.

###### Financial Anxiety

We adapted one item from the Financial Anxiety Scale (Archuleta et al., 2013) with parents rating the extent to which they felt anxious about their financial situation during the pandemic using a 5-point scale (*1 = Not at all, 5 = Extremely).*

###### Financial Difficulty

Parents rated how difficult it had been to afford necessities using a 5-point scale (*1 = Not at all difficult, 5 = Extremely difficult).* Parents rated difficulty affording food, rent or mortgage, utilities and bills, things needed for children, and other items. We computed a mean score for responses for the four items named herein (α = 0.941).

##### Marital Domain

###### Marital Quality

Marital quality was measured with three items from the Couple Satisfaction Index (Funk and Rogge, 2007) probing marital satisfaction, marital affection, and marital conflict (e.g., *“In general, how satisfied are you with your relationship with your spouse/partner?”*), to which parents responded using a 6-point scale (*0 = Never, 5 = All time time).* Responses to the marital conflict item were reverse-scored, and a mean score was computed for analyses (α = 0.696).

###### Satisfaction With Partner’s Help

Using a 7-point scale (*1 = Very Dissatisfied, 7 = Very satisfied)*, parents rated their satisfaction levels with two aspects of their partner’s help: Taking care of children, and doing household work (*r* = 0.633, *p* < 0.001). A mean score was calculated with higher values reflecting greater satisfaction with partner’s help.

##### Psychosocial Assets Domain

###### Active Coping Skills

We measured parents’ coping skills using the Brief Resilient Coping Scale (BRCS; Sinclair and Wallston, 2004). Using a 5-point scale, parents rated the extent to which statements accurately described their behavior and actions on four items (e.g., *“I look for creative ways to alter difficult situations,” 1 = Does not describe me at all, 5 = Describes me very well).* We computed a mean score of the four items with higher scores reflecting better coping skills (α = 0.686).

###### Emotion Regulation – Cognitive Reappraisal

We measured cognitive reappraisal using the four items of the Cognitive Reappraisal subscale of the Emotion Regulation Questionnaire (ERQ; Gross and John, 2003). Parents rated the extent to which they agreed with statements about emotion regulation (e.g., *“When I’m faced with a stressful situation, I make myself think about it in a way that helps me stay calm”)* using a 7-point scale (*1 = Strongly disagree, 7 = Strongly Agree).* We computed a mean score from the 4 items with higher scores indicating greater use of cognitive reappraisal (α = 0.819).

##### Child Characteristics Domain

###### Child Gender

Child gender was measured as a parent’s response to the following item, *“Please specify the gender of the child that you’ll be focusing on.”* Response options included “female,” “male,” “non-binary,” “other – please specify,” and “decline to answer.” None of the parents selected “non-binary” or “other – please specify” as gender identities for their focal child. Two parents declined to answer; focal child gender was marked as missing for those parents.

###### Child Age

Child age was measured as a parent’s response to the following item, *“How old is the child that you will be focusing on?”.*

###### Child Temperament – Emotionality

Three items from the Emotionality, Activity, and Sociability (EAS) Temperament Questionnaire (Buss and Plomin, 1984) emotionality subscale were used to measure children’s emotional temperament. Parents rated how characteristic each item was of the focal child (e.g., *“My child gets upset easily,” 1 = Not at all, 5 = Highly)*. All three items were used to compute a mean score with higher scores reflecting having a more emotional child (α = 0.83).

##### Covariates Included in Prediction of Family Functioning Profiles

Given the role of pre-existing sociodemographic factors in impacting both parents’ and children’s responses to the early stages of the pandemic (Hawkins, 2020; Hooper et al., 2020; Feinberg et al., 2021), we controlled for parent race and ethnicity, parental educational level, parent gender, number of children in the household, and presence of an essential worker living in the household when examining predictors of family functioning profiles.

###### Race/Ethnicity

Parents were asked to report their race and ethnicity with the following response options: “White/Caucasian,” “Black/African American,” “Hispanic or Latinx,” “Asian/Asian-American/Pacific Islander,” “Native American/American Indian,” Middle Eastern/North African,” “biracial or multiracial (please describe),” “other (please specify),” and “decline to state.” Because of the preponderance of parents identifying as “White/Caucasian,” this was treated as a bivariate (0/1) measure, while recognizing the inherent assumptions and limitations of this approach.

###### Educational Level

Parents were asked to report the highest level of education that they had completed. Response options included “high school,” “some college,” “community college,” “bachelor’s degree,” “graduate degree,” and “other (please specify).” Higher values represent higher education level.

###### Parent Gender

Parents’ gender was measured as their response to the following question, *“What gender do you most identify with?”.* Response options included “female,” “male,” “non-binary,” “other – please specify,” and “decline to answer.” No parents selected “non-binary” or “other – please specify.” One parent declined to answer; gender was marked as missing data for that parent.

###### Number of Children in Household

Parents were asked to report the number of children currently living in the household.

###### Essential Worker Living in Household

Parents reported on whether any adults living in the household were an essential worker (e.g., healthcare worker, delivery person, store worker, security person, worked in building maintenance). Responses included “yes” and “no” which were dummy-coded into 1 and 0, respectively.

#### Predicting Child Adjustment From Family Functioning Profiles

##### Child’s Psychosocial Adjustment

At both T1 and T2, the parent report version of the Strengths and Difficulties Questionnaire (SDQ; Goodman, 1997) for children ages 2–17 years was used to measure children’s psychosocial adjustment during the pandemic. Parents completed the SDQ at both timepoints with T1 subdomain scores used as covariates in respective analyses. The measure includes 25 items describing children’s behavioral strengths and difficulties with the parent rating each item on a 3-point scale (*1 = not true, 2 = somewhat true, 3 = certainly true)*. Of note, we did not include the five items measuring the peer problems subdomain as these items pertain to physical interactions with others, which would not have been applicable for most children during the early months of shelter-in-place. Consequently, parents rated 20 items, delineated into four subdomains: prosocial behavior (five items, α = 0.756, 0.774, at T1, T2, respectively), emotional problems (five items, α = 0.750, 0.703), conduct problems (five items, α = 0.581, 0.517), and hyperactivity problems (five items, α = 0.736, 0.774). Additionally, we calculated total difficulties, which is the sum of the emotional, conduct, and hyperactivity problem subdomains (α = 0.807, 0.808).

##### Covariates Included in Prediction of Child Adjustment From Profiles

We included child age, child gender, parent race/ethnicity, child EAS temperament scores, and child SDQ psychosocial adjustment scores as covariates. All covariates were measured at T1.

###### Child Temperament – Activity

The EAS Temperament Questionnaire (Buss and Plomin, 1984) was used to measure children’s active temperament, which was used as a covariate in analyses with SDQ hyperactivity problems as the outcome. Specifically, we used the three items from the activity subscale wherein parents rate how characteristic each item is of their child (e.g., *“My child is always on the go,” 1 = Not at all, 5 = Highly)*. All three items were used to compute a mean score with higher scores reflecting having a more active child (α = 0.771).

###### Child Temperament – Emotionality

As previously described, the EAS Temperament Questionnaire (Buss and Plomin, 1984) was used to measure children’s emotional temperament, which was used as a covariate in analyses with SDQ emotional problems as the outcome.

### Analytic Plan

Data analyses consisted of four stages: (1) data pre-processing; (2) LPA to identify profiles of family functioning; (3) antecedents predicting latent profiles; and (4) child outcomes at T2 predicted from latent profiles at T1. Data pre-processing and preliminary analyses were performed in SPSS, version 26. Mixture modeling for LPA and associated analyses were performed in MPlus, version 8.5. LPA was used rather than other person-centered analyses as it is compatible with continuous indicators (whereas latent class analysis requires nominal indicators) and is a probabilistic, model-based approach that affords flexible modeling of latent profile covariates and distal outcomes (whereas cluster analysis requires identical scales across indicators and does not permit flexible auxiliary models; Hagenaars and McCutcheon, 2002).

#### Data Pre-processing

Variables were reviewed for univariate normality, identifying and transforming univariate outliers, and assessing for systematic missingness. Univariate normality was assessed using skew and kurtosis within absolute value of 3 and 10, respectively (Howell, 1997) in addition to visual inspection of histograms. SDQ emotional problems score was negatively skewed at both timepoints and was subsequently log-transformed. We considered outliers to be any value that exceeded +3 SDs from the mean. We identified four outliers for T1 SDQ conduct problems, one outlier for T2 SDQ conduct problems, one outlier for T1 SDQ total difficulties, and one outlier for T1 cognitive reappraisal. These values were winsorized while retaining rank order.

At T1, missingness ranged from 0.200 to 6.900% across demographic and key study variables. At T2, missingness was 3.700% across key study variables. There was 52% attrition from T1 to T2 (*n* = 234). Of note, when the study was launched at T1, parents were not told that there would be a follow-up assessment (as the pandemic was not projected to continue) but were asked to indicate their interest in future study participation. At T2, only parents who had indicated their interest were contacted for follow-up. Attrition between timepoints reflects families who either did not consent to being contacted for future research or who chose not to complete the follow-up survey. In preliminary analyses, we performed Little’s Missing Completely at Random test (MCAR; Little, 1988) to assess if data were missing completely at random both within and between study timepoints. Additionally, we performed independent samples *t*-tests and chi square tests of independence between participants who only participated at T1 only and those who participated at both timepoints to probe for group differences on demographic and key study variables (i.e., indicator, antecedent, and distal outcome variables) to assess if attrition was systematically related to any measured demographic characteristic.

Little’s MCAR test provided evidence that data may be missing completely at random for T1 variables, χ^2^(178) = 190.998, *p* = 0.239, and for T2 variables, χ^2^(68) = 26.158, *p* = 0.999. For attrition analyses, Little’s MCAR test provided evidence that data may be missing completely at random, χ^2^(246) = 102.603, *p* = 0.999. Additionally, chi square tests of independence and independent samples *t*-tests – both with multiple comparisons adjustment – did not find any significant differences between T1 and T2 participants on demographic and key study variables. Overall, our findings suggest that data at both timepoints were missing completely at random and attrition was not due to systematic differences between participants. For the auxiliary predictor analysis, we performed multiple imputation with 10 datasets to account for missingness and to avoid listwise deletion. However, given the large amount of attrition between timepoints, we did not impute data for T2 variables in the child outcome analyses (*n* = 219).

#### Profiles of Family Functioning

Mixture modeling with maximum likelihood estimation were performed in MPlus version 8.5. We used an iterative process of fitting k + 1 classification models and examination of multiple indices combined with theoretical interpretation to determine the best class solution (Collins and Lanza, 2009; Nylund-Gibson and Choi, 2018). Specifically, entropy, Akaike information criteria (AIC), sample size adjusted Bayesian information Criteria (adjusted BIC) (Symonds and Moussalli, 2011), the Lo, Mendal Ruben test (LMR; Lo et al., 2001), and the bootstrapped likelihood ratio test (BLRT; Nylund et al., 2007) were used to determine best fit (Nylund-Gibson and Choi, 2018). For AIC and adjusted BIC, lower values indicate a relatively better fitting model (Symonds and Moussalli, 2011). Entropy is a measure of how well the indicators are distinguishing classes with values approaching 1 indicating more clear delineation of classes (Celeux and Soromenho, 1996). There is not a consensus for a specific cutoff value for entropy; however, it is generally agreed upon that an entropy value above 0.60 in combination with average latent class probabilities above 0.80 indicates distinguishable classes (Spurk et al., 2020). Thus, entropy was considered in conjunction with other model fit indices. The LRM and the BLRT tests evaluate improvement between class solutions with a significant *p*-value suggesting significant improvement in fit for the k + 1 solution (Lo et al., 2001; Nylund et al., 2007). We also reviewed the average latent class probabilities for class assignment wherein probabilities should be 0.80 or higher (Nylund et al., 2007). Finally, LPA assumes that identified classes are real and theoretically meaningful; thus, theoretical rationale was also used in determining the best fitting solution (Raudenbush, 2005).

#### Auxiliary Predictors of Latent Profile Membership

After identifying latent profiles, we next examined what factors distinguished latent profile membership. We followed Vermunt’s three-step approach with the manual method (Vermunt, 2010; Asparouhov and Muthén, 2014). In step 1, we used mixture modeling to fit the class solution. In step 2, following an “error-in-variable” schema, we saved the estimated conditional probabilities to be used as an estimated average classification error of class assignment to minimize class shifting and estimation error. Finally, in step 3, we performed multinomial logistic regression wherein classification error is taken into account to prevent class shifting due to antecedent variables and to minimize bias. Specifically, financial impact, marital dynamics, psychosocial assets, and child characteristics domain variables were entered into the multinomial logistic regression, all while controlling for sociodemographic variables. The thriving profile was used as the reference group for analysis.

##### Covariates for Auxiliary Predictors

We controlled for parent race/ethnicity, parental educational level, parent gender, number of children in the household, and presence of an essential worker living in the household in the auxiliary predictor analysis.

#### Latent Profile Prediction of Child Adjustment

We used the manual three-step approach to examine the extent to which family functioning profiles prospectively predicted child functioning later in the pandemic controlling for T1 covariates. In step 3, we performed an omnibus Wald chi-square test of mean equality among the family functioning profiles followed by pairwise comparisons to identify specific outcome differences between latent profiles (Asparouhov and Muthén, 2014; Nylund-Gibson et al., 2019). We fit five models with the best LPA solution predicting children’s SDQ prosociality, emotional problems, conduct problems, hyperactivity, and total difficulties at T2, respectively.

##### Covariates for Distal Outcomes

Child age, child gender, parent race/ethnicity, and corresponding T1 SDQ scores were included as covariates. Additionally, we controlled for T1 EAS – emotionality when examining T2 SDQ emotional problem outcomes, and we controlled for T1 EAS – activity when examining T2 SDQ hyperactivity outcomes.

## Results

### Preliminary Analyses

Descriptive statistics for all key study measures are in Table 1, and zero-order correlations are in the Supplementary Table S1.

**TABLE 1:** Descriptive statistics for key study variables.

	*M*(*SD*)	Skew	Kurtosis	Missing (%)
**Family Thriving Indicators**				
Parent-Child Relationship Quality	3.202(0.819)	0.155	0.003	0.900
Parental Satisfaction	4.530(1.675)	–0.326	–1.087	4.900
Parental Efficacy	3.170(1.148)	0.253	–0.399	5.300
Parent Positive Adjustment	4.085(0.817)	–0.186	–0.264	6.900
Child’s Emotional Well-Being	3.470(0.991)	–0.182	–0.519	6.200
**Antecedents of Family Functioning**				
*Financial Domain*				
Financial Anxiety	2.480(1.206)	0.548	–0.615	0.000
Financial Difficulty	1.546(0.871)	1.697	2.134	0.000
Income Per Capita	3.127(1.430)	0.249	–0.231	4.700
*Marital Domain*				
Satisfaction with Partner’s Help	5.246(1.616)	–0.720	–0.535	0.200
Marital Quality	3.297(1.017)	–0.427	–0.282	0.000
*Psychosocial Assets Domain*				
Cognitive Reappraisal_*a*_	4.833(1.010)	–0.390	0.108	6.400
Active Coping Skills	3.886(0.598)	–0.381	0.246	6.400
*Child Characteristics Domain*				
Child Emotionality	3.113(1.040)	0.042	–0.756	5.300
Child Age	7.019(4.064)	0.862	–0.126	0.900
Child Gender	1.540(0.499)	–0.154	–1.985	1.100
**Distal Child Outcomes**				
Prosociality	6.062(2.310)	–0.265	–0.522	3.700
Emotional Problems_*b*_	0.612(0.199)	0.135	–0.885	3.700
Conduct Problems_*a*_	2.131(1.550)	0.542	–0.241	3.700
Hyperactivity	4.825(2.547)	0.036	–0.656	3.700
Total Difficulties	9.431(4.940)	0.440	–0.384	3.700

*N = 449.*

*Subscript “a” denotes a winsorized variable. Subscript “b” denotes a log-transformed variable.*

### Latent Profile Analysis

We ran LPA with one to five classes (see Table 2 for fit statistics) and determined the four-profile model to be the best fitting solution due to the following reasons. First, the 3-class and 4-class solution yielded comparable entropy values (0.769, 0.775) that were superior to the 5-class solution. Second, the 4-class solution had lower information criteria values as compared to the 2-class and 3-class solution (albeit, higher than the 5-class). Third, both the LMR and BLRT indicated the 4-class solution was significantly better fitting than the 3-class solution. The LMR (but not the BLRT) suggested that the 5-class solution was not significantly better fitting than the 4-class solution. The average latent class probabilities for the 4-class solution were all above 0.80 (0.835–0.904) but this threshold was not met for the 5-class solution (0.790–0.889). Finally, from a theoretical perspective, the 4-class vs. 5-class solutions appeared to be capturing similar patterns of family functioning across all indicators with the 4-class solution providing more conceptually clear and distinct family functioning patterns (for 5-class solution; see Supplementary Figure S1). Thus, when considering both empirical and conceptual criteria, we determined the 4-class solution to be the best fitting.

**TABLE 2:** Fit indices for latent profile solutions.

	2 Class	3 Class	4 Class	5 Class
AIC	5898.060	5780.398	**5695.266**	5682.867
Adjusted BIC	5912.994	5800.933	**5721.402**	5714.604
Entropy	0.828	0.775	**0.769**	0.737
LMR LRT	2 vs. 1 value: 382.640, *p* < 0.001	3 vs. 2 value: 126.217, *p* < 0.001	**4 vs. 3 value: 94.552, *p* < 0.001**	5 vs. 4 value: 23.750, *p* = 0.070
BLRT	2 vs. 1 value: 393.083, *p* < 0.001	3 vs. 2 value: 129.662, *p* < 0.001	**4 vs. 3 value: 97.132, *p* < 0.001**	5 vs. 4 value: 24.399, *p* = 0.013

*N = 449.*

*LMR LRT, Lo-Mendell-Rubin Adjust Likelihood Ratio Test; BLRT, bootstrapped likelihood ratio test.*

**FIGURE 2:**
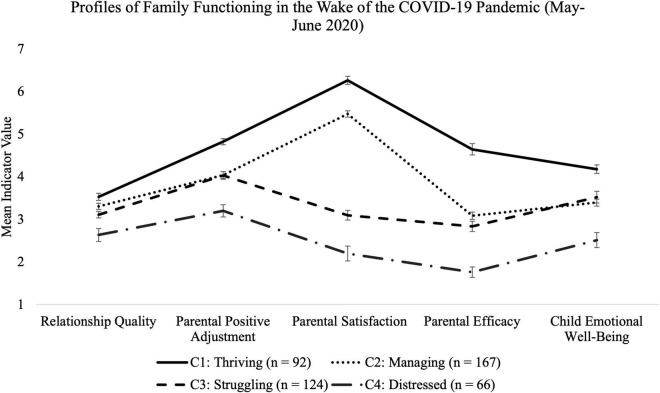
Plot comparisons of estimated means and standard errors of four different family functioning profiles during the early stages of the COVID-19 pandemic (May–June 2012). High values indicates more positive outcomes for all family thriving indicators. A 4-class solution was determined to be the best-fitting solution with profiles identified in the plot key.

Within the 4-class solution (Figure 2; exact means in Supplementary Table S2), class 1 represents a “thriving” class (C1, *n* = 92, 20.489% of sample) wherein families reported improved parent–child relationship quality and the highest levels of parental satisfaction, parental efficacy, parent positive adjustment, and child emotional well-being. Class 2 (C2, *n* = 167, 37.194% of sample) represents families who were “managing” in that they had slightly improved parent–child relationship quality and moderately high levels of parental satisfaction, parental efficacy, parental positive adjustment, and child emotional well-being; however, all indicators were at lower levels compared to C1: Thriving. Class 3 (C3, *n* = 124, 27.617% of sample) represents families who were “struggling” as reflected in their low parental efficacy and parental satisfaction scores, despite parent-child relationship quality remaining the same as from before the pandemic, and despite having reported moderately high levels on parental and child mental health indicators. Finally, Class 4 (C4, *n* = 66, 14.699% of sample) represents “distressed” families that exhibited the lowest levels of functioning across all indicators.

We performed a one-way analysis of variance (ANOVA) to test for latent profile mean differences in each of the indicators (i.e., parent–child relationship quality, parental satisfaction, parental efficacy, parent positive adjustment, and child emotional well-being). There were significant mean differences between profiles on parent-child relationship quality, *F*(3,198.033) = 21.296, *p* < 0.001, parental efficacy, *F*(3,201.733) = 342.994, *p* < 0.001, parental satisfaction, *F*(3,421) = 820.475, *p* < 0.001, parent positive adjustment, *F*(3,414) = 83.240, *p* < 0.001, and child emotional well-being, *F*(3,192.860) = 60.493, *p* < 0.001 (Supplementary Table S2). *Post hoc* Bonferroni pairwise comparisons found that C1: Thriving had significantly higher levels of parental efficacy, parental satisfaction, parent positive adjustment, and child emotional well-being compared to all other profiles. Additionally, C4: Distressed has significantly lower levels of all family functioning indicators compared to all other profiles. Also, Profiles C2: Managing had significantly higher scores than C3: Struggling on parental efficacy and satisfaction.

### Auxiliary Predictors of Latent Profile Membership

Next, we included financial, psychosocial assets, marital, and child factors as auxiliary predictors of the latent profiles while controlling for sociodemographic factors (Vermunt, 2010; Nylund-Gibson et al., 2019). To determine which factors distinguished thriving families from other profiles, we set C1: Thriving as the reference group in the multinomial logistic regression. All variables from each domain were included in one model. Relative risk ratios (RRR) were calculated for all logistic regression estimates (Table 3).

**TABLE 3:** Multinomial logistic regression coefficients for financial, marital, psychosocial assets, and child predictors for latent profile membership.

	C2 versus C1: Managing Relative to Thriving		C3 versus C1: Struggling Relative to Thriving		C4 versus C1: Distressed Relative to Thriving	
			
Predictors	RRR*(SE)*	*Z*	RRR*(SE)*	*Z*	RRR*(SE)*	*Z*
**Demographic Variables**						
Essential Worker in Household	**2.710(0.518)**	**−1.924*^t^***	2.038(0.471)	1.512	**4.860(0.668)**	2.365*
Parental Education Level	1.413(0.218)	–1.589	1.175(0.179)	0.901	1.487(0.249)	1.593
Parent Gender	0.447(0.608)	–0.962	0.767(0.541)	–0.490	0.307(0.897)	–1.316
Race and Ethnicity	0.775(0.580)	–0.004	1.052(0.428)	0.096	1.155(0.723)	0.200
Number of Children in Home	1.200(0.310)	–0.440	1.332(0.260)	1.102	1.252(0.332)	0.679
**Financial Domain**						
Financial Anxiety	1.306(0.273)	–0.979	**1.852(0.237)**	2.594**	**2.243(0.319)**	2.531*
Financial Difficulty	1.576(0.545)	–0.836	1.067(0.410)	0.159	0.932(0.559)	–0.125
Income Per Capita	1.349(0.255)	–1.173	1.169(0.213)	0.734	1.143(0.286)	0.470
**Psychosocial Assets Domain**						
Cognitive Reappraisal	**0.512(0.290)**	−2.311*	**0.555(0.271)**	−2.170*	**0.287(0.389)**	−3.208**
Active Coping Skills	1.636(0.436)	–1.127	0.759(0.410)	–0.671	0.538(0.530)	–1.169
**Marital Domain**						
Satisfaction with Partner’s Help	0.751(0.228)	–1.257	**0.611(0.206)**	−2.384*	**0.493(0.247)**	−2.858**
Marital Quality	0.801(0.277)	–0.800	0.852(0.256)	–0.628	0.778(0.308)	–0.816
**Child Domain**						
Child Emotionality	**3.343(0.429)**	−2.811**	**2.433(0.343)**	2.593**	**5.094(0.511)**	3.189**
Child Age	0.915(0.089)	–1.357	0.940(0.056)	–1.106	1.020(0.075)	0.269
Child Gender	1.560(0.445)	1.001	1.195(0.404)	0.441	1.035(0.570)	0.062

*N = 449. Significance values are provided in bold.*

^t^p < 0.10, *p < 0.05, **p < 0.01.

For sociodemographic covariates, families that had an essential worker living in the household had a significantly greater likelihood of being in C4: Distressed (RRR = 4.263, *p* = 0.024) in comparison to C1: Thriving. Parent’s gender, educational level, race/ethnicity, and number of children in the household did not significantly predict profile membership.

For the financial domain, families with increased financial anxiety had a significantly greater likelihood of being in C3: Struggling (RRR = 1.852, *p* = 0.009) and a significantly greater likelihood of being in C4: Distressed (RRR = 2.243, *p* = 0.011) in comparison to C1: Thriving. Financial difficulties and income per capita did not significantly predict profile membership.

For the psychosocial assets domain, parents with higher cognitive reappraisal scores had significantly lower likelihood of being in C2: Managing (RRR = 0.511, *p* = 0.021), C3: Struggling (RRR = 0.555, *p* = 0.030), and C4: Distressed (RRR = 0.287, *p* = 0.001) in comparison to C1: Thriving. Active coping skills did not significantly predict profile membership.

For the marital domain, parents with greater satisfaction with partner’s help had a significantly lower likelihood of being in C3: Struggling (RRR = 0.611, *p* = 0.017) and had a lower likelihood of being in C4: Distressed (RRR = 0.493, *p* = 0.004) in comparison to C1: Thriving. Marital quality did not significantly predict profile membership.

For the child characteristics domain, having a more temperamentally emotional child decreased the likelihood of being in the C1: Thriving profile compared to the in C2: Managing (RRR = 3.343, *p* = 0.005), C3: Struggling (RRR = 2.433, *p* = 0.010), and C4: Distressed (RRR = 5.094, *p* = 0.001) profiles. Child gender and age did not significantly predict profile membership.

### Child Outcomes Predicted From Latent Profiles

As shown in Table 4, the omnibus Wald test of mean equality indicated that there were significant differences between latent profiles on children’s prosociality [χ^2^(3) = 9.250, *p* = 0.026], emotional problems [χ^2^(3) = 23.143, *p* < 0.001], conduct problems [χ^2^(3) = 9.412, *p* = 0.024], and total difficulties [χ^2^(3) = 77.386, *p* < 0.001] at T2, but not hyperactivity [χ^2^(3) = 3.435, *p* = 0.329]. Accounting for T1 SDQ scores, T1 LPA profiles predicted T2 child adjustment over and above the stability of child adjustment. For prosociality, scores linearly decreased from C1: Thriving to C4: Distressed, but pairwise comparisons did not find specific significant differences between profiles for SDQ prosociality scores. For children’s emotional problems, pairwise comparisons revealed that C1: Thriving had significantly lower SDQ emotional problem scores compared to all other profiles at T2 (*p*’s = 0.001–0.006). For children’s conduct problems, C1: Thriving had significantly lower SDQ conduct problem scores compared to C3: Struggling (*p* = 0.035). For children’s total difficulties, C1: Thriving had significantly lower SDQ total difficulties scores compared to all other profiles at T2 (*p*’s < 0.001).

**TABLE 4:** Family functioning profiles predicting distal child outcomes at Time 2 (September–October 2020).

	C1: Thriving (*n* = 38)	C2: Managing (*n* = 88)	C3: Struggling (*n* = 65)	C4: Distressed (*n* = 28)	Mean (SE)

	*T2 SDQ – Prosociality*				
Omnibus Test	χ^2^(3) = 9.250*				
C1: Thriving	—				06.44 (0.354)
C2: Managing	χ^2^(1) = 0.021	—			06.16 (0.272)
C3: Struggling	χ^2^(1) = 0.024	χ^2^(1) = 0.002	—		05.87 (0.311)
C4: Distressed	χ^2^(1) = 0.145	χ^2^(1) = 0.001	χ^2^(1) = 1.065	—	05.70 (0.411)

	*T2 SDQ – Emotional Problems*				

Omnibus Test	χ^2^(3) = 23.143***				
C1: Thriving	—				00.88 (0.090)
C2: Managing	χ^2^(1) = 10.728** C1 < C2	—			01.27 (0.093)
C3: Struggling	χ^2^(1) = 7.717** C1 < C3	χ^2^(1) = 0.020	—		01.45 (0.119)
C4: Distressed	χ^2^(1) = 22.769*** C1 < C4	χ**^2^(1) = 3.608*^t^* C2 < C4**	χ^2^(1) = 2.432	—	01.89 (0.123)

	*T2 SDQ – Conduct Problems*				

Omnibus Test	χ^2^(3) = 9.412*				
C1: Thriving	—				01.32 (0.188)
C2: Managing	χ^2^(1) = 1.139	—			02.13 (0.163)
C3: Struggling	χ^2^(1) = 4.439* C1 < C3	χ^2^(1) = 0.819	—		02.35 (0.216)
C4: Distressed	χ**^2^(1) = 2.965*^t^* C1 < C4**	χ^2^(1) = 0.016	χ^2^(1) = 1.542	—	02.67 (0.214)

	*T2 SDQ – Hyperactivity*				

Omnibus Test	χ^2^(3) = 3.435				
C1: Thriving	—				04.12 (0.324)
C2: Managing	—	—			04.63 (0.259)
C3: Struggling	—	—	—		05.13 (0.286)
C4: Distressed	—	—	—	—	05.63 (1.306)

	*T2 SDQ – Total Difficulties*				

Omnibus Test	χ^2^(3) = 77.386***				
C1: Thriving	—				06.65 (0.504)
C2: Managing	χ^2^(1) = 60.656*** C1 < C2	—			09.07 (0.543)
C3: Struggling	χ^2^(1) = 66.284*** C1 < C3	χ^2^(1) = 0.814	—		10.21 (0.642)
C4: Distressed	χ^2^(1) = 49.561*** C1 < C4	χ^2^(1) = 0.770	χ**^2^(1) = 3.412*^t^* C3 < C4**	—	12.30 (1.032)


*N = 219. Significance values are provided in bold.*

^t^p < 0.10, *p < 0.05, **p < 0.01, ***p < 0.001.

## Discussion

Leveraging person-centered analyses, the current study identified distinct profiles of family functioning early in the COVID-19 pandemic; examined financial, marital, psychosocial, and child predictors associated with those family functioning profiles; and predicted subsequent child adjustment from those profile as the pandemic persisted. Within this sample of relatively advantaged and protected families, we found a distinct pattern of Thriving families during shelter-in-place, which the LPA distinguished from three other family functioning profiles: Managing, Struggling, and Distressed families. Also as expected, indicators across multiple domains distinguished the Thriving profile from the other three profiles, and particularly from the Distressed profile, and children in Thriving families had better psychological adjustment 6 months after shelter-in-place restrictions due to the pandemic began in the United States.

While there has been deserved focus on the challenges experienced by both parents and children during the pandemic, our findings suggest that many families were resilient. Two to three months after the COVID-19 pandemic severely affected living conditions, just over 20% of sampled families were Thriving, exhibiting improved parent-child relationship quality, excellent mental and emotional health in both parent and child, and high parenting confidence and efficacy. This family functioning profile reflects similar findings of families with children being brought together during shelter-in-place across multiple countries (Chavez et al., 2021). The largest proportion of sampled families (∼37%) were Managing, characterized as having slightly improved or unchanged parent-child relationship quality coupled with parents and children reporting positive adjustment and good mental health during shelter-in-place, high parent satisfaction in their role as a parent, and moderate efficacy.

Therefore, more than 50% of parents thought that their families were doing well; conversely, almost as many parents did not share this experience. About a quarter of the sampled families (∼28%) were characterized as Struggling. Interestingly, although their parent-child relationship quality, parental positive adjustment, and child health were comparable to the Managing profile, Struggling parents reported slightly lower parental efficacy and substantially lower parental satisfaction. Thus, parents in the Struggling families could be seen as overly self-critical; they were coping reasonably well, but not as well as they thought they could or should be. Finally, about 15% of the sampled families in this study were Distressed. The early stages of the pandemic detrimentally impacted the well-being of these families, which were characterized by worsened parent-child relationships, parent and child psychological maladjustment to pandemic conditions, and low levels of parental satisfaction and efficacy.

Considering our examination of auxiliary predictors of these family functioning profiles, parent’s use of cognitive reappraisal and child emotionality consistently distinguished Thriving families from Managing, Struggling, and Distressed families, and other specific factors within the examined domains provided further distinctions. The breadth of predictors across domains underscores the multidimensional nature of risk and resilience (Masten, 2015; Masten and Motti-Stefanidi, 2020), and suggests multiple possible levers of intervention to assist families.

### Financial Domain Predictors

Of note, our sample was relatively economically advantaged and stable; very few families had experienced income or job loss at the time of data collection. Within this sample, actual financial conditions (income per capita, financial difficulties) did not distinguish family profiles, yet anxiety about future financial security distinguished both Struggling and Distressed families from Thriving families. Prior research with more socioeconomically diverse samples has shown that pandemic-related family hardship undermined family well-being (Gassman-Pines et al., 2020; Chen et al., 2021; Feinberg et al., 2021; Schmeer et al., 2021). As the profiles did not differ in actual financial resources, financial worry may be another stressor that permeated across the socioeconomic spectrum during this period of societal economic uncertainty. Possibly, Struggling and Distressed families may have had fewer financial resources in reserve, leading parents to feel concerned about their economic stability if pandemic public health regulations (closures of non-essential businesses) and associated impacts (furloughs, job loss) continued for longer than projected. From a family stress model perspective (Conger and Elder, 1994; Conger and Conger, 2002), this subjective or emotional aspect of financial stress may uniquely contribute to parental negative affect, marital tension, and harsh parenting with downstream effects on family functioning and child adjustment.

### Marital Domain Predictors

Interestingly, although overall marital quality did not distinguish between profiles, parents who were more satisfied with their partner’s contributions to childcare and household chores had higher odds of being in the Thriving family profile, relative to the Struggling and Distressed profiles. This parallels prior examinations of work-family balance strategies during shelter-in-place and associated family well-being (Shockley et al., 2020; Yavorsky et al., 2021). Satisfaction with partner’s help mediates associations between distribution of domestic labor and marital quality (Stevens et al., 2001, 2005), and having a partner who contributed to domestic labor may have helped Thriving parents to cope more effectively with the pandemic conditions (Prime et al., 2020). Couples therapy that targets partners’ ability to self-advocate and proactively plan for how they will transition as a family to pandemic conditions may better equip couples to communicate the ways in which they need support with domestic labor and to mutually create a plan for division of household tasks (Stanley and Markman, 2020). Looking forward, telecommuting and location flexibility will likely remain even after the pandemic subsides (Ozimek, 2020), and our findings highlight the critical role of equitable division of labor in fostering the ability of families to cope well with this changed landscape of employment and decreased distinction between work-life and home-life (Shockley et al., 2020). Employers and policymakers could also adjust workplace policies to better support families with two parents with expanded, paid family leave (Crompton, 2006; Lewis, 2009). Given the prospective association between family thriving and children’s later adjustment, workplace policies that support work-family balance that enable more egalitarian domestic labor strategies may also have downstream effects on promoting children’s mental health and adjustment.

### Psychosocial Assets Predictors

Parents who reported more use of cognitive reappraisal had significantly higher likelihood of being classified as Thriving in comparison to all other family functioning patterns, whereas parents’ active coping skills did not profile membership. Coping is inherently linked to actions made in response to adversely stressful situations whereas cognitive reappraisal is an emotion regulation strategy that can be utilized across a spectrum of emotions, not just in negative situations or experiences (Compas et al., 2014). Cognitive reappraisal may better equip parents to both effectively manage their stress, anger, and sadness during shelter-in-place *and* enable them to maintain a positive outlook, and recognize and experience positive emotions (e.g., love, joy, awe) during a time of increased family contact. Positive reappraisal – a form of cognitive reappraisal in which the goal is to increase positive emotion – can both decrease distress and increase positive affect even during negative or stressful contexts (McRae et al., 2012; Shiota and Levenson, 2012).

Cognitive reappraisal could enable parents to find “the silver lining” in pandemic conditions, which may benefit the entire family. Pandemic studies have found that parents’ cognitive reappraisal is associated with reduced personal distress and parenting stress (Preuss et al., 2021), and that parents with better overall emotion regulation have children who were less stressed in reaction to COVID-19 (Shorer and Leibovich, 2020). Through use of cognitive reappraisal, parents may be modeling effective emotion regulation strategies for their children (Gottman et al., 1996; Eisenberg et al., 1998). In relation to the Prime et al. (2020) framework of family well-being during COVID-19, cognitive reappraisal may facilitate meaning-making processes wherein the family as a whole can “make sense” of the pandemic by incorporating their experience into a coherent worldview, jointly minimize catastrophic thinking, and reframe the pandemic as manageable (Walsh, 2015).

### Child Characteristic Domain Predictors

Notably, child emotionality was one of our most robust predictors of family functioning during the early stages of COVID-19 as it distinguished all family functioning profiles from Thriving. That is, families that had a child who was less temperamentally emotional were more likely to be thriving and coping well with pandemic conditions. This finding builds upon burgeoning research exploring the role of children’s high emotionality in contributing to parent and child maladjustment during the pandemic (Zhou et al., 2021) and undermining structured parenting for parents experiencing high levels of distress. From a determinants of parenting perspective (Belsky, 1984; Bornstein, 2016), highly emotional children may erode a parent’s capacity to sensitively respond to and soothe a child during the stressful, and at times, scary aspects of the pandemic. Children’s dispositional emotional instability – a correlate of emotionality – has been associated with greater parental burnout and exhaustion (Vigouroux and Scola, 2018), which may contribute and coalesce into overall family dysfunction (Mikolajczak et al., 2018).

However, within a differential susceptibility framework (Ellis et al., 2011), temperamentally difficult children may be more responsive to positive change in their psychosocial environments. Indeed, in a randomized, controlled parenting intervention, temperamentally emotional children particularly benefitted from improved parenting behavior (Scott and O’Connor, 2012). During the early stages of the pandemic, promoting parents’ confidence and self-compassion can enhance their feelings of self-efficacy with direct effects on alleviating parenting stress and downstream effects on children’s increased emotional well-being and decreased emotional lability and negativity (Morelli et al., 2020). Consequently, providing practical support and education to parents for assisting with the specific needs of highly emotional children could assist with their ability to effectively manage family functioning during the pandemic (e.g., Tuning into Kids^®^; Havighurst et al., 2020).

### Implications of Family Functioning for Children’s Ongoing Adjustment to the Pandemic

Our results also indicate that how a family initially responded to and coped with COVID-19 conferred lasting implications for children’s ongoing adjustment. Families who thrived during the early months of the pandemic had children who maintained their well-being over the ensuing months, exhibiting more prosocial behavior and fewer emotional, conduct, and total behavioral problems in comparison to other family functioning profiles during Fall 2020. Conversely, children in Distressed families had poor mental and emotional health during shelter-in-place and continued to exhibit the most behavioral difficulties during Fall 2020. Despite Struggling parents being more self-critical, their children’s adjustment was commensurate with that of children in Managing families. Fostering Struggling parents’ self-compassion could help them attend to and address their potentially overly critical evaluations of their competencies.

Following life course history (Elder, 1998), the ongoing COVID-19 pandemic represents a defining, global event with lasting effects on all persons experiencing it. The pandemic will likely have the most intense, enduring effect on children’s developmental trajectories as COVID-19 continues to pervade and disrupt their family, social, and academic spheres (Benner and Mistry, 2020). Amidst anecdotal reports of children’s increased rates of disruptive behavior and worsening socioemotional health in the classroom (Leonhardt, 2022) and the American Academy of Pediatrics declaring a national state of emergency in children’s mental health (AAP-AACAP, 2021), our findings underscore the importance of providing families with support and resources to better prepare children for the transition back to school and extracurricular activities. Policies that provide adequate financial and healthcare assistance, flexible and family-supportive workplaces, and targeted marital and psychosocial interventions are all ways to promote family thriving that may buffer children against pandemic stressors and promote better psychosocial adjustment, both as COVID-19 continues and in its aftermath.

### Implications for Intervention

The beginning of the COVID-19 pandemic introduced change and upheaval in nearly every aspect of family’s daily lives. Yet, some families coped and managed quite well while others struggled. Our findings build upon emerging research that multifactorial interventions targeting multiple aspects of the family system (i.e., spousal relationship, each individual parent, individual child) and the family as a whole are needed to best support families. From a social cognitive perspective, interventions that target parents’ self-compassionate beliefs and re-orient parents to recognizing their personal strengths may bolster parenting competence and emotion regulation with subsequent benefit to their children’s coping (Morelli et al., 2020). Interventions targeting parent’s cognitive reappraisal may be particularly fruitful avenues for promoting overall family well-being, as this was a robust predictor of family functioning in our study, has been found to alleviate parenting stress in a randomized control trial (Preuss et al., 2021) and has been found to promote adults’ emotional well-being during the pandemic (Wang et al., 2021). Transdiagnostic approaches, such as dialectical behavior therapy, may be effective in addressing parent’s emotion dysregulation while promoting parenting competencies (Zalewski et al., 2018). The Unified Protocol for the Transdiagnostic Treatment of Emotional Disorders in Children (UP-C) piloted “Coping with COVID” – a parent-led, therapist-assisted, cognitive behavioral teletherapy – and produced promising, preliminary results. Participating families reported heightened parental self-efficacy, children’s improved emotional functioning, and decreased family distress, particularly for children with emotional difficulties (Guzick et al., 2022). While further research is warranted to determine the effectiveness of “Coping with COVID,” these preliminary results are encouraging for teletherapy targeting family functioning.

### Contextualizing the Findings: Important Caveats

Our sample was relatively homogenous and advantaged, and our findings cannot be presumed to be reflective of how other U.S. sociodemographic communities have experienced the COVID-19 pandemic. The extent to which pandemic disruptions impacted families must be contextualized within systemic inequities of race, ethnicity, and economic security that have affected family responses to and impacts from shelter-in-place (Prime et al., 2020). Racially and ethnically minoritized and marginalized communities, and economically disadvantaged families, have experienced disproportionately more adverse employment, scholastic, and social stressors resulting from the pandemic (Chen et al., 2021) and are more likely to have lower-wage “essential” jobs, leading to increased risk for contracting COVID-19 (Hawkins, 2020). Our finding that having an essential worker in the household significantly increased the likelihood that a family would be classified as Distressed relative to Thriving provides a small glimpse into how socioeconomic disparity undermines family functioning during an unprecedented disaster. It is essential for future research to include more diverse samples to better understand what promotes family thriving across the sociodemographic spectrum during a global health crisis.

### Limitations and Strengths

Our study includes both weaknesses and strengths. We relied on a single-parent reporter for parent, child, and family measures at both timepoints, which may lead to shared method variance and inflated associations. Although we collected retrospective reports of pre-pandemic conditions, we did not collect data prior to COVID-19, limiting our ability to assess the full extent to which family functioning was impacted or changed by pandemic conditions. It is likely that some families experienced strained parent-child relationships or poor mental health prior to COVID-19 and these dynamics were maintained during shelter-in-place. Our use of convenience sampling may have contributed to our sample being quite homogeneous and not reflective of national demographics, and as previously discussed, this limits the generalizability of our findings. Future research with more diverse samples and with single-parent households is warranted. Finally, we had substantial attrition at Time 2 which resulted in underrepresentation of the Thriving and Distressed family functioning profiles. The decreased sample size at Time 2 limited our statistical power to identify differences in child adjustment across family profiles.

However, our study is notable in using a multidimensional, strengths-based approach in examining family functioning and to probe into how families may be functioning well during the pandemic, adding nuanced insight into multiple dimensions of family life early in the pandemic. By leveraging person-centered analyses, we were able to model the heterogeneity in families’ initial responses to COVID-19 and associated regulations, enabling us to examine how these factors co-occurred within the family system, and providing unique insight into family well-being as a comprehensive whole. Another major strength of our study is the longitudinal design during key timepoints: in the wake of the pandemic (Spring 2020) and at the beginning of youths’ second school year during the pandemic (Fall 2020). Measuring more than one timepoint affords better understanding of the acute and enduring effects of the pandemic on family adjustment and helps further our understanding about family-level factors most pertinent to children’s adjustment throughout the course of the pandemic (Wade et al., 2020).

## Future Directions and Conclusion

Future research should explore whether the latent profiles we identified were stable or if families shifted into different types of functioning as the pandemic continued, and the implications of such family-level processes for children’s continued adjustment. Understanding which families continued to flourish and what helped them maintain their thriving can inform intervention efforts. Importantly, replication in more diverse samples is essential, particularly examining families who were more severely financially impacted early in the pandemic, to further our understanding of what supports thriving in all families during the pandemic.

As the COVID-19 pandemic persists and potentially becomes endemic, it is crucial for developmental science to study the associated risk and protective factors that impact family well-being. Our study reveals that there are multiple pathways of risk and resilience for family functioning during this exceptional time in global history. These pathways indicate tractable targets for psychosocial intervention that are amenable to individual and family counseling. Utilizing these potential levers to promote mental health in families languishing during the COVID-19 pandemic could promote resilience to this continuing health crisis and future crises, thereby protecting children’s well-being.

## Data Availability Statement

The raw data supporting the conclusions of this article will be made available by the authors, on request to PH, pdhastings@ucdavis.edu.

## Ethics Statement

The studies involving human participants were reviewed and approved by University of California, Davis Institutional Review Board. The participants provided their written informed consent to participate in this study.

## Author Contributions

LP developed the study concept for the current investigation and drafted the manuscript. LP and MM performed the survey design and data collection under the supervision of PH. LP performed the data analysis and interpretation under the supervision of PH. PH and MM provided the critical revisions. All the authors contributed to the study design and approved the final version of the manuscript for submission.

## Conflict of Interest

The authors declare that the research was conducted in the absence of any commercial or financial relationships that could be construed as a potential conflict of interest.

## Publisher’s Note

All claims expressed in this article are solely those of the authors and do not necessarily represent those of their affiliated organizations, or those of the publisher, the editors and the reviewers. Any product that may be evaluated in this article, or claim that may be made by its manufacturer, is not guaranteed or endorsed by the publisher.
