# Supplementary material for: Family Thriving During COVID-19 and the Benefits for Children’s Well-Being
Source: Front Psychol. 2022 May 12;13:879195. doi: 10.3389/fpsyg.2022.879195 (PMC9135131; doi:10.3389/fpsyg.2022.879195)
Supplement: Supplementary file 1 [file Data_Sheet_1.pdf]

Supplementary Information

Table S1 provides zero-order correlations for key study variables, delineated by family thriving indicators, antecedents of family functioning, and distal child outcomes. Table S2 provides results of one-way analysis of variance for family functioning indicators between latent profiles. Figure S1 presents the 5-class solution from latent profile analysis, which was deemed to be inferior to the 4-class solution.

	1.	2.	3.	4.	5.	6.	7.	8.	9.	10.	11.	12.	13.	14.	15.	16.	17.	18.	19.	20.
Family Thriving Indicators																				
1. Parent-Child Relationship Quality	–																			
2. Parental Satisfaction	.32***	–																		
3. Parental Efficacy	.22***	.61***	–																	
4. Parent Positive Adjustment	.23***	.44***	.54***	–																
5. Child Emotional Well-Being	.22***	.31***	.41***	.37***	–															
Antecedents of Family Functioning																				
<u>*Financial Domain*</u>																				
6. Financial Anxiety	.03	-.22***	-.22***	-.34***	-.13**	–														
7. Financial Difficulty	.09^t	-.09^t	-.09^t	-.16***	-.15**	.63***	–													
8. Income Per Capita	.04	.08	.09^t	.08	.12*	-.30***	-.46***	–												
<u>*Marital Domain*</u>																				
9. Satisfaction with Partner's Help	.18***	-.28***	.22***	-.28***	.22***	-.19**	-.20***	-.23***	–											
10. Marital Quality	.09^t	.21***	.24***	.34***	.15**	-.15**	-.11***	.05	.41***	–										
<u>*Psychosocial Assets Domain*</u>																				
11. Cognitive Reappraisala	.15**	.28***	.28***	.29***	.11*	.02	.09^t	-.05	-.03	.08	–									
12. Active Coping Skills	.19***	.29***	.25***	.30***	.15**	.04	.03	.01	.08	.15**	.55***	–								
<u>*Child Characteristics Domain*</u>																				
13. Child Emotionality	-.17***	-.23***	-.33***	-.28***	-.34***	.05	-.04	-.01	-.16***	-.11*	-.23***	-.19***	–							
14. Child Age	.09*t*	.05	.13**	.18***	-.20***	-.04	-.01	-.05	-.01	.04	.07	.07	-.14**	–						
15. Child Gender	-.04	-.02	-.01	-.03	-.05	-.05	-.06	.05	-.01	-.01	-.04	-.08	.13**	.02	–					
Distal Child Outcomes																				
16. Prosociality	.16*	.20**	.06	.01	.29***	-.14^t	-.11	-.04	.01	.05	.04	.15*	-.21**	-.03	-.21**	–				
17. Emotional Problems$_b$	-.08	-.28***	-.33***	-.33***	-.34***	.20**	.14*	-.02	.20**	-.16*	-.06	.01	.31***	.01	-.06	-.11	–			
18. Conduct Problems$_a$	-.18**	-.25***	-.22**	-.25***	-.19**	.25***	.17*	-.03	.17*	-.12^t	-.04	-.07	.34***	-.14*	.14*	-.40***	.37***	–		
19. Hyperactivity	-.14*	-.21**	-.16*	-.25***	-.22**	.17*	.13^t	.01	.23***	-.28***	-.06	-.11	.28***	-.22**	.13^t	-.30***	.37***	.51***	–	
20. Total Difficulties	-.16*	-.30***	-.29***	-.36***	-.33***	.26***	.19**	-.02	.25***	.25***	-.07	-.09	.38***	-.15*	.09	-.33***	.73***	.75***	.84***	–

te. N = 449. Subscript "a" denotes a winsorized variable. Subscript "b" denotes a log-transformed variable. $^t p < .10$ $*p < .05$ $** p < .01$ $*** p < .001$.

Table S2

Means, Standard Errors, and One-Way Analysis of Variance of in Family Functioning Indicators Between Latent Profiles

	Class 1: Thriving		Class 2: Managing		Class 3: Struggling		Class 4: Distressed			
Indicator Variable	*M*	*SE*	*M*	*SE*	*M*	*SE*	*M*	*SE*	*F*	*df*
Parent-Child Relationship Quality	3.527[a]	0.084	3.305[ab]	0.073	3.109[b]	0.077	2.632[c]	0.151	21.296***	3, 198.033
Parental Satisfaction	6.262[a]	0.097	5.473[b]	0.074	3.096[c]	0.114	2.197[d]	0.174	820.475***	3, 421
Parental Efficacy	4.644[a]	0.133	3.084[b]	0.086	2.834[c]	0.120	1.759[d]	0.122	342.994***	3, 201.733
Parent Positive Adjustment	4.823[a]	0.073	4.039[b]	0.080	4.029[b]	0.089	3.199[c]	0.144	83.240***	3, 414
Child Emotional Well-Being	4.177[a]	0.101	3.393[b]	0.082	3.520[b]	0.137	2.513[c]	0.176	60.493***	3, 192.860

Note: Means in a row with different superscripted letters differed significantly at $p < .05$ in post hoc Bonferroni pairwise comparisons (i.e., a > b > c > d). $^{***}p < .001$.
