# Supplementary figures and images for: Family Thriving During COVID-19 and the Benefits for Children’s Well-Being
Source: Front Psychol. 2022 May 12;13:879195. doi: 10.3389/fpsyg.2022.879195 (PMC9135131; doi:10.3389/fpsyg.2022.879195)

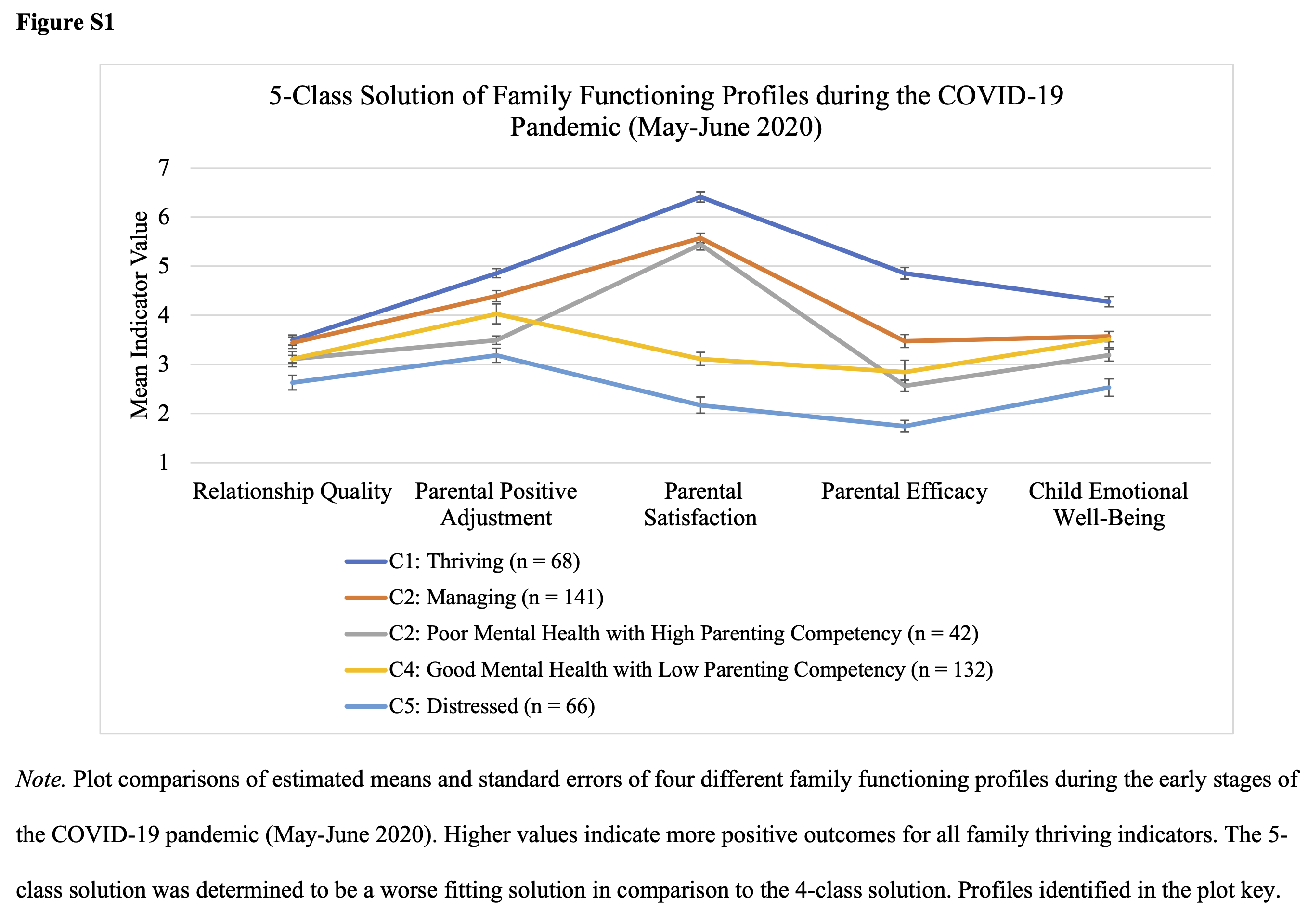

Supplement: Supplementary file 2 [file Image_1.tiff]
